# Feasibility and success rates of response enhancing strategies in a stepwise prevention program for cardiometabolic diseases in primary care

**DOI:** 10.1186/s12875-020-01293-9

**Published:** 2020-11-06

**Authors:** Ilse F. Badenbroek, Marcus M. J. Nielen, Monika Hollander, Daphne M. Stol, Roderik A. Kraaijenhagen, Niek J. de Wit, François G. Schellevis

**Affiliations:** 1 Netherlands Institute for Health Services Research (NIVEL), P.O. Box 1568, 3500 BN Utrecht, the Netherlands; 2 JULIUS CENTER FOR HEALTH SCIENCES AND PRIMARY CARE, University Medical Center Utrecht, P.O. Box 85500, 3508 GA Utrecht, the Netherlands; 3 NDDO Institute for Prevention and Early Diagnostics (NIPED), Naritaweg 70, 1043 BZ Amsterdam, The Netherlands; 4 Department of General Practice & Elderly Care Medicine, EMGO Institute for health and care research, VU University Medical Center, Van der Boechorststraat 7, 1081 BT Amsterdam, the Netherlands

**Keywords:** Cardiovascular diseases, Primary prevention, Health risk assessment, General practice, Patient participation rates

## Abstract

**Background:**

Prevention programs for cardiometabolic diseases (CMD), including cardiovascular disease, diabetes mellitus and chronic kidney disease are feasible, but evidence for the cost-effectiveness of selective CMD prevention programs is lacking. Response rates have an important role in effectiveness, but methods to increase response rates have received insufficient attention. The aim of the current study is to determine the feasibility and the success rate of a variety of response enhancing strategies to increase the participation in a selective prevention program for CMD.

**Methods:**

The INTEGRATE study is a Dutch randomised controlled trial to assess the effectiveness and cost-effectiveness of a stepwise program for CMD prevention. During the INTEGRATE study we developed ten different response enhancing strategies targeted at different stages of non-response and different patient populations and evaluated these in 29 general practices.

**Results:**

A face-to-face reminder by the GP increased the response significantly. Digital reminders targeted at patients with an increased CMD risk showed a positive trend towards participation. Sending invitations and reminders by e-mail generated similar response rates, but at lower costs and time investment than the standard way of dissemination. Translated materials, information gatherings at the practice, self-management toolkits, reminders by telephone, information letters, local media attention and SMS text reminders did not increase the response to our program.

**Conclusions:**

Inviting or reminding patients by e-mail or during GPs consultation may enhance response rates in a selective prevention program for CMD. Different response-enhancing strategies have different patient target populations and implementation issues, therefore practice characteristics need to be taken into account when implementing such strategies.

**Trial registration:**

Dutch trial Register number NTR4277. Registered 26 November 2013.

**Supplementary Information:**

**Supplementary information** accompanies this paper at 10.1186/s12875-020-01293-9.

## Background

Cardiometabolic diseases (CMD), including cardiovascular disease, diabetes mellitus and chronic kidney disease exercise increasing pressure on the healthcare costs. Because of the modifiable nature of many risk factors, 80% of cardiometabolic diseases can be prevented [1]. Selective prevention programs for CMD, targeting an apparently healthy population with a stepwise program in order to select only those at high risk, seem a promising and cost-effective strategy [1, 2]. With this approach risk-reducing interventions can be targeted at patients at high risk. However, up to now, no convincing evidence has been provided that selective prevention programs for CMD are cost-effective [3]. An important aspect in the effectiveness of a prevention program is the response rate [4], as studies have shown a wide variation in participation [2, 3, 5]. Mapping response rates and identifying methods to improve them has received little attention so far. If we could increase participation in prevention programs, their effectiveness on population level would increase simultaneously.

Several studies have been performed to gain more insight into the characteristics of non-responders in prevention programs for CMD [5–14]. Non-responders are reported to be more often male, smoke more often and being of younger age [5, 7, 8, 10–14]. A lower socio-economic status (SES) and a migrant status is also reported to be related to non-response [5, 7, 9, 10, 15]. If these groups could be reached with targeted response enhancing strategies, this could lead to an increased participation.

Various studies explored strategies to enhance response rates in preventive and screening programs in general [16, 17] and for prevention programs for CMD specifically [18]. Commonly used methods are reminders by letter or telephone, face-to-face reminders by a physician, providing educational material and publicity through different media. With the aforementioned characteristics of non-responders taken into account, more advanced response-enhancing strategies are developed to specifically reach the underperforming groups. Invitations or reminders by e-mail [19] or SMS text messages [20] might be attractive for the younger population. The provision of a toolkit for self-testing already resulted in an enhanced uptake for cervical cancer screening [17]. Patients from lower SES groups and migrants may benefit from culture specific information meetings and translated questionnaires [21].

Successful response enhancing strategies may also be guided by specific preferences of non-responders. During the INTEGRATE study [22] we assessed attitudes towards different response enhancing strategies amongst non-responders. Most non-responders would reconsider participation if they would receive a face-to-face invitation by their own GP, if the awareness of the program would be enhanced through media exposure or if more informative information would be offered. However, the feasibility and success rates of these strategies are yet to be determined in clinical practice.

The design of the INTEGRATE study [23] made it possible to develop and to evaluate response enhancing strategies in the same study population. Here we report on the feasibility and the success rates of a variety of response enhancing strategies to increase the participation in a selective prevention program for CMD.

## Methods

### INTEGRATE study

The INTEGRATE study is a stepped-wedge randomized controlled trial that ran from 2014 to 2017. The aim of the INTEGRATE study is to assess the cost-effectiveness of a stepwise CMD prevention program. In 37 participating general practices, all listed patients between 45 and 70 years old without CMD, hypertension or hypercholesterolemia were invited to participate in the prevention program. Patients were randomly allocated to an intervention group or a waiting list control group that was invited for the program after 1 year. To minimize the workload in the GP practices, both the intervention and the waiting list control group were enrolled gradually using different time slots (intervention group 1 and 2 in April and June 2015 and control group 1 and 2 in April and June 2016). The first step was selecting eligible patients, who were subsequently asked to complete a risk estimation score (RS) consisting of seven risk factors for CMD (age, gender, smoking status, body mass index (BMI), waist circumference, family history of type II diabetes mellitus and cardiovascular disease). Eligible patients received a personal invitation letter from their own GP to complete the RS online. All invitation letters included a short summary of the instruction in English, Turkish and Arab. All patients who did not respond within 2 weeks received a reminder for the online RS and additionally a paper version of the RS and a return envelope.

Patients with a low score on the RS received (online) tailored lifestyle advice. Patients with a high score on the RS (increased risk patients) were advised to visit their GP for additional measurements to complete their risk profile, followed by a tailored lifestyle advice and/or treatment if indicated. Further details of the design of the INTEGRATE study are described elsewhere [23].

### Study population

All strategies were implemented during the follow-up phase of the trial, during which the waiting list control groups were invited for participation in the prevention program. The patients in the waiting list group were randomly assigned to a subgroup within the same practice, the ‘strategy group’ (exposed to a response enhancing strategy) or to the ‘standard method group’ (approached as described in the previous section).

**Fig. 1:**
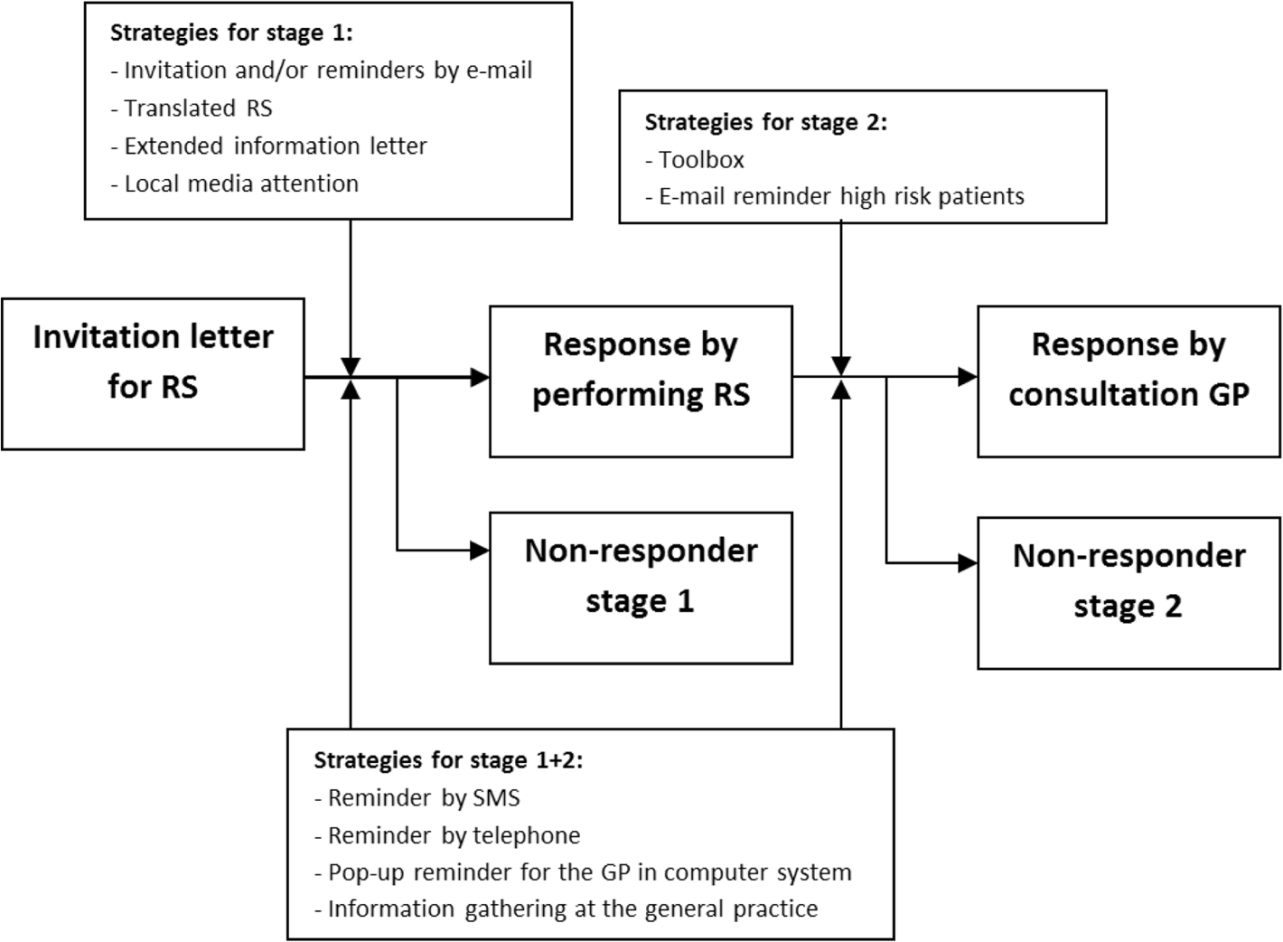
Stages of response and timing response enhancing strategies

Different strategies for increasing response were targeted at non-responders at different stages of the prevention program, stage 1 and stage 2. Non-responders stage 1 were the non-responders who did not respond to the online or paper RS, non-responders stage 2 were the non-responders with an increased risk score on the RS who did not contact their GP. Some strategies were aimed at both types of non-responders (stage 1 + 2). An overview of the different strategies timed at the different stages is shown in Fig. 1.

In 29 of the 37 participating practices in the INTEGRATE study one or more response enhancing strategies was implemented; in 8 practices it was not feasible to arrange a strategy within the set timeframe. The different strategies were allocated in consultation with the participating practices, guided by specific practice characteristics such as patient population (number of people of low SES and/or migrant status), the percentage of patients of whom the e-mail address or mobile phone number was known in the practice, the availability of support staff and the individual preferences of the GP or practice nurse.

### Response enhancing strategies

Based on the literature and a survey among first phase non-responders [22] we developed ten different response enhancing strategies. Taking the suggestions of the first phase non-responders into consideration, we selected strategies using a pop-up reminder in the computer system of the GP, extended information letters and information gatherings at the general practice, among others. The background and implementation of these strategies, their timing, types of non-responders (stage 1, stage 2 or stage 1 + 2) and the number of practices and patients involved are described in Table 1 and Fig. 1.

**Table 1:** Characteristics of response enhancing strategies

	Stage	Strategy group(N)	Standard method group (N)	Practices involved	Procedure
**Standard method**					Invitation letter for online RS by post, after 2 weeks reminder letter + paper version RS by post. A short recap in English, Turkish and Arab in both letters.
**1. Invitation and/or reminders by e-mail**	Stage 1	328 (patients with known e-mail address)	124 (patients with known e-mail address)	2	Group a (*N* = 124): comparison group (standard method)Group b (*N* = 105): invitation by post and a reminder by e-mail; Group c (*N* = 117): invitation by e-mail and a reminder by post; Group d (*N* = 106): invitation and reminder by e-mail.
**2. Translated RS form**	Stage 1	4604	N/A	10	Translated versions of the RS form in English, Turkish and Arab were added to the reminder letter.
**3. Extended information letter**	Stage 1	337	337	1	Extension of the information letter emphasizing the importance of participating and uncovering CMD and risk factors.
**4. Local media attention**	Stage 1	381	232	2	Articles about the prevention program and the importance of participating were placed in local newspapers.
**5. Reminder by SMS**	Stage 1 + 2	73 (patients with known mobile number)	194	2	Four weeks after the paper invitation the GP sent SMS text reminders to patients with a known mobile number, inviting patients to fill in the RS and to make an appointment when this was advised.
**6. Reminder by telephone**	Stage 1 + 2	34 (random sample)	N/A	1	Practice assistant called the patients after 4 weeks to inform if the RS was filled in and to offer an appointment when the patients this was advised.
**7. Pop-up reminder GP in computer system**	Stage 1 + 2	341	340	2	A pop-up message would appear in the computer system of the GP if the patient file was opened. The GP could inform the patient about the RS during a regular consultation.
**8. Information gathering at the general practice**	Stage 1 + 2	545	N/A	2	The GP practice organized an information gathering to help patients fill in the RS and/or to offer additional measures at the general practice. Invitation through invitation letter with recap translated in English, Turkish and Arab.
**9. Self-management toolkits**	Stage 2	174 (patients with high score on RS)	N/A	6	We offered patients a free toolkit containing a blood pressure device and fingerstick for cholesterol and Hba1c. Patients received online tailored lifestyle advice and were advised to consult their GP if increased blood pressure and/or elevated serum levels were measured.
**10. E-mail reminder increased risk patients**	Stage 2	112 patients with high score on RS	127 patients with high score on RS	4	We sent patients a reminder by e-mail to contact their GP for an appointment.

### Outcome measures

The success rate was based on the response rates of either step of the program. The response to the RS was defined as the percentage of patients who completed the RS (either online or on paper) of the total number of patients who received an invitation to do so. Participation in stage 2 of the prevention program was defined as the number of patients with an increased risk score on the RS who visited the GP according to the case report forms, electronic medical record or self-reported in the study questionnaire.

To determine the feasibility of the response enhancing strategies we estimated the time investment and additional costs per strategy, which were subsequently expressed in categories (low/average/high) for an average sized practice population for 1 full-time GP (*n* = 2095 patients). The definition of time investment was low when the time spent was half or less than half the time compared to the standard method. Time investment was high when twice as much time or more was needed. Costs were low when the costs were half or less compared to the costs for the standard method, the costs were high when the costs were twice as high or more.

### Statistical analysis

Descriptive analyses of all measurements were performed. Univariate multilevel logistic regression analyses were used to compare the differences in response rates between those exposed to the response enhancing strategies and those exposed to the standard method. Crude odds ratios and 95% confidence intervals were reported. Stata version 15 was used for all statistical analyses.

The response for the RS showed a 3% seasonal variation (June vs. April), most likely due to a lower response during the summer holiday. This difference was seen in the two intervention groups as well as in the waiting list control groups. We therefore adjusted the response rates in the groups invited in June by adding 3% to the response at the RS.

## Results

All ten response enhancing strategies turned out to be feasible in the setting of the INTEGRATE study; 29 general practices implemented one or more strategies. Response rates for the RS in the standard method groups was on average 33%, ranging from 16 to 48% between practices. From all patients filling in the RS 38% turned out to be at increased risk, 38% of these patients visited the GP.

We sent a translated version of the RS (strategy 2) to 4604 patients; only 12 (0.3%) completed translated RSs were returned (2 in English, 7 in Turkish and 3 in Arab).

Mobile numbers were known for only 73 of the 188 patients eligible to receive a reminder via SMS (strategy 5). They received a SMS reminder 4 weeks after the invitation letter. Sixteen patients (22%) filled in the RS after receiving the SMS, whereas 11 patients from the standard method group (15%) spontaneously filled in the RS in these 4 weeks. Nine out of the sixteen patients who filled in the RS after an SMS had an increased risk. None of the patient who received an SMS showed up for a GP consultation.

Of the 34 patients who were scheduled for a reminder by telephone (strategy 6), 11 patients (32%) could not be reached by telephone after two attempts within office hours. Among those who could be reached, 13 patients (38%) completed the RS of which 4 patients had an increased risk and reported to have already made an appointment with the GP. The remainder 10 patients (29%) indicated not to be interested in participating.

The two information gatherings (strategy 8) were scarcely attended: of the 545 invited patients only 3 patients showed up from the first practice and 2 patients at the meeting in the second practice (response rate of 0.9%).

Self-management toolkits (strategy 9) were offered for free to 174 patients at increased risk. 33 toolkits were ordered, 22 patients completed their risk profiles with self-executed measures, resulting in 6 patients with an increased risk who were given the advice to visit their GP of which 1 patient actually visited.

To determine the effectiveness of the other response enhancing strategies (strategy 1, 3,4,7 and 10) we compared the response rates of the RS (stage 1) and participation of stage 2 of the prevention program between the strategy groups and the standard method groups. The results of these analyses are shown in Table 2.

**Table 2:** Response rates strategy and standard method groups for response enhancing strategies

	Strategy group			Standard method group			Difference in response (OR with 95%CI)	
	Invited (n)	Response RS (%) ^a^	Consultations GP (n (%)) ^b^	Invited (n)	Response RS (%) ^a^	Consultations GP (n (%)) ^b^	Stage 1	Stage 2
**1. Postal invitation + Email reminder (b)**	105	45%	N/A	124	48%	N/A	0.89 [0.53–1.50]	N/A
**1. Email invitation + Postal reminder (c)**	117	50%	N/A	124	48%	N/A	1.12 [0.68–1.86]	N/A
**1. Email invitation + Email reminder (d)**	106	58%	N/A	124	48%	N/A	1.49 [0.89–2.51]	N/A
**3. Extended information letter**	337	39%	N/A	337	45%	N/A	0.79 [0.58–1.08]	N/A
**4. Local media attention**	381	37%	N/A	232	38%	N/A	0.93 [0.67–1.31]	N/A
**7. Pop-up reminder GP in computer system**	341	37%	16 (36%)	338	33%	3 (10%)	1.22 [0.51–2.93]	4.63 [1.15–18.67]
**10. E-mail reminder increased risk patients**	124	N/A	57 (46%)	144	N/A	52 (36%)	N/A	1.51 [0.92–2.46]

^a^ Response rates for RS shown are a corrected for a 3% lower response in groups invited in June

^b^ number and percentage of patients with increased risk at RS that consulted GPAbbreviations: *RS* Risk score

We found a significant increase in the participation rate for stage 2 of the prevention program when the GP received a pop-up reminder when opening the patient’s electronic medical record (strategy 7) (OR 4.63 [1.15–18.67]).

Although invitations and reminders by e-mail (strategy 1) did show a positive trend in improving participation (OR 1.51 [0.92–2.46]), none of the implemented combinations of invitations and/or reminders by e-mail showed a significant increase in response amongst the non-responders at stage 1. However, we observed in practices who implemented strategy 1 that, independently of the invitation method, the response rate for the RS amongst patients with an e-mail address known to the GP practice was statistically significantly higher than amongst patients without a known e-mail address (49% vs. 32%, *p* = 0.000, not shown in table), demonstrating that this variable is strongly associated with response.

Sending an e-mail reminder to increased risk patients (strategy 10) also had a positive, but not statistically significant, effect on the number of patients who consulted their GP (OR 1.46 [0.86–2.48]).

We found no effect of local media attention (strategy 4) on the response rates. Those who received an extended information letter (strategy 3) had a lower response rate to the RS and a lower participation rate to stage 2 of the program, although this difference was not statistically significant (OR 0.79 [0.58–1.08]).

Table 3 shows an overview of the response enhancing strategies which we implemented during this study, with their target group, an estimation of the costs and time investment required and implementation challenges experienced in the participating practices. The time spent on the standard method was on average 10 h per practice, with the costs involved estimated at € 750 per average sized practice. The required time for the different strategies ranged from 4 h to 47 h, the costs ranged from € 0 to € 2025. More details about the actual time investment and the calculation of the costs can be found in Additional file 1. E-mail reminders and/or invitations are low in costs and require limited time investment, however do require computer skilled personnel and a substantial part of the patients whose e-mail address is known in the practice. All response enhancing strategies had their specific target population and implementation issues, making different strategies more suitable for different practices depending on practice characteristics and patient population. Therefore, the feasibility of the different strategies strongly depends on the circumstances and characteristics of the practice.

**Table 3:** Target population, costs, time investment and implementation issues of different response enhancing strategies

Response enhancing strategy	Target population	Time ^a^	Costs ^b^	Implementation issues
**Standard method**	Total	Average	Average	Printing and folding invitation letters is time consuming
**1. Invitation and/or reminders by e-mail**	Young	Low	Low	E-mail address often unknown, computer skilled personnel necessary
**2. Translated RS**	Migrant	Average	High	Costly and time consuming when not targeted
**3. Extended information letter**	Total	Average	Average	Overload of information may provide opposite effect
**4. Local media attention**	Total	Average	Average	Willingness (local) media channels required
**5. Reminder by SMS**	Young	Average	Average	Mobile phone numbers sometimes unknown, computer skilled personnel necessary
**6. Reminder by telephone**	Total	High	Average	Motivated personnel necessary, unavailability of patients during office hours
**7. Pop-up reminders GP computer system**	Total	High	Average	Manually entering reminders is time consuming, motivated GP necessary, time consuming during consultation hours
**8. Information gathering general practice**	Low SES or migrant	Average	High	Motivated personnel necessary, targeted population difficult to reach
**9. Self-management toolkits**	Young	Average	High	Self-management skills patients necessary, preferably with feedback results to GP
**10. E-mail reminder increased risk patients**	Young	Average	Average	Feedback from online RS to GP necessary, computer skilled personal necessary

^a^ Time investment for standard method for average sized general practice (*n* = 2095 patients) was 10 hLow: 5 h or less, Average: 6–19 h, High: 20 h or more

^b^ Costs investment for standard method for average sized general practice (*n* = 2095 patients) were €750

Low: €375 or less, Average: €376 to €1499, High: €1500 or more

Abbreviations: *RS* Risk score

## Discussion

### Main findings

In this study we evaluated different strategies to increase the participation rates in a selective prevention program for CMD in primary care. Using a pop-up reminder, integrated in the computer system of the GP, increased the participation to our prevention program significantly. We also found a positive trend in the response rate when using e-mail invitations and reminders, a method that requires little time investment and the costs. Translated materials, information gatherings at the practice, self-management toolkits, reminders by telephone, extended information letters, local media attention and SMS text reminders did not increase the response to our program.

### Strengths and limitations

This is the first study to implement and evaluate different response enhancing strategies within the context of an RCT evaluating the effectiveness of a CMD prevention program. For most response enhancing strategies we were able to compare the effect of the intervention to a control group from the same practice. The design of the INTEGRATE study made it possible to develop different response enhancing strategies with input from the target population and implement the strategies in the same study population. One of the most important limitations of our study is that due to the many strategies tested, the number of patients in the strategy and standard method groups were small, which influenced the statistical power and therefore the robustness of the results. Nevertheless, the results provide a useful insight into the more and less effective strategies.

### Comparison with current literature

The positive effect of face-to-face reminders by the GP is in line with the results of a meta-analysis of Cheong et al. [18], who found reminding physicians to invite patients for cardiovascular screening to be effective. The time investment for entering pop-ups in the computer system, however, is high so this method would be best suited for practices that work with a computer system that has an option to create pop-ups automatically. This method also requires that the GP is motivated to address the cardiovascular risk when a patient consults for different reasons.

To the best of our knowledge, this is the first study on the effect of reminders and invitations by e-mail on the response rates in prevention programs for CMD. This strategy can be easily implemented, the extra time investment and the costs are low. The different combinations of e-mail invitations and/or reminders for the RS we implemented did not significantly increase the response. Nevertheless, approaching patients by e-mail resulted in comparable response rates as the standard method, but at lower costs and with less time investment. A remarkable observation was that patients whose e-mail address was known in the GP practice, were more likely to participate regardless the method of approaching. This might be explained by the health care consumption pattern of these patients. Those with a high health care utilization have more frequent contacts with their GP, which makes it more likely that their e-mail address is recorded. In addition, patients who have a better relationship with their GP might feel more compelled when their GP sends them a personal invitation for a RS. In contrast, patients with a tendency to avoid health care have no regular contacts with their GP and may therefore not be inclined to fill in a RS.

Sending an additional reminder by e-mail to patients with an increased risk showed a positive trend on the response rate at stage 2 of the program. Both this additional reminder and the face-to-face reminders target patients who scored high on the RS, these patients showed to have an increased risk for CMD and also showed to be willing to enter the program by participating in stage 1. Nevertheless, on average only 1 in 3 patients with an increased risk visited their GP. These non-responders are an interesting target group for they could potentially benefit most from a prevention program.

The effectiveness of reminders by telephone was low in this study. This is in line with the non-response analysis we performed earlier, where 78% of the non-responders reported that they would not reconsider participation when approached by telephone [22]. Letter and telephone reminders showed significantly higher response rates in cancer screening programs [16, 17] and prevention programs for CMD, some of them in a study population with a low SES [24–26]. It is a possibility that reminders by telephone are more effective when they can be targeted more specifically.

The effect of the extended information letter showed a negative effect on the response rate, although this effect was not statistically significant. It is possible that an information ‘overload’ decreased the motivation to participate. Previous studies showed heterogeneous results for extended educational material and public information campaigns [17, 27]. The extended information letter that we used in our study was not personalised, a potentially stimulating factor that might be addressed more with a face-to-face reminder [5, 9, 28].

Translated materials and information gatherings, strategies specifically targeted at the migrant and/or low SES population, were unsuccessful even though the costs were high. These strategies cannot be recommended for other prevention programs.

Part of the suggested response enhancing strategies given by the first phase non-responders during our earlier survey did not function in real condition. A possible explanation for this gap is that motivation for prevention and health behaviour are more complex than we, and the non-responders themselves, had estimated and therefore proves difficult to influence.

## Conclusions

Patients identified at increased CMD risk are the optimal target group for response enhancing strategies, this could be achieved with the installation of pop-ups in the GPs’ computer systems to facilitate personal reminders. Invitation by e-mail for a prevention program is feasible at relatively low costs and time investment, though further research to confirm these findings is warranted. Finally, enhancing response in prevention programs for CMD is a difficult and complex process. There is no ‘one size fits all’ response enhancing protocol for all general practices. Population and practice characteristics need to be carefully considered to select successful recruitment strategies.

## Supplementary Information

**Additional file 1. **Detailed overview of different response enhancing strategies**.** p.p. = per patient, min = minute(s), RS = risk score * Average practice size of 2095 patient, approximately 375 patients eligible for prevention program. ** If 19% of patients with increased risk on RS order the box (17 patients).

## Data Availability

The datasets used and/or analysed during the current study are available from the corresponding author on reasonable request.

## Abbreviations

CMD: Cardiometabolic diseases
BMI: Body mass index
GP: General practitioner
min: Minute(s)
p.p.: Per patient
RS: Risk score
SES: Socio-economic status
