# Supplementary material for: Feasibility and success rates of response enhancing strategies in a stepwise prevention program for cardiometabolic diseases in primary care
Source: BMC Fam Pract. 2020 Nov 6;21:228. doi: 10.1186/s12875-020-01293-9 (PMC7648376; doi:10.1186/s12875-020-01293-9)
Supplement: Supplementary file 1 — Additional file 1. Detailed overview of different response enhancing strategies. p.p. = per patient, min = minute(s), RS = risk score * Average practice size of 2095 patient, approximately 375 patients eligible for prevention program. ** If 19% of patients with increased risk on RS order the box (17 patients). [file 12875_2020_1293_MOESM1_ESM.docx]

| **Response enhancing strategy** | **Time investment** | | | | **Costs** | | | |
| --- | --- | --- | --- | --- | --- | --- | --- | --- |
|  | **Fixed** | **Variable** | **Total per practice *** | **Category** | **Fixed** | **Variable** | **Total per practice *** | **Category** |
| ***Standard method*** | *Standard time =  Writing + formatting + mailing letters +:* ***4 hours*** | *Standard time =  Printing + handling letters:* ***1 min p.p.*** | *10 hours* | *Average* |  | *Standard costs:  Printing materials and postal costs:* ***€ 2 p.p.*** | *€ 750* | *Average* |
| **1. Invitation and/or reminders by e-mail** | Formatting e-mails and mailing: 4 hours |  | 4 hours | Low |  |  | € - | Low |
| **2. Translated RS** | Standard time | Standard time + Extra printing and handling translations:  1 min p.p. | 16 hours | Average | Translation costs: € 500 | Costs standard method + extra postal costs: € 1 p.p. | € 1625 | High |
| **3. Extended information letter** | Standard time + Formatting extra information letter: 2 hours | Standard time + Printing and handling extra letter: 1 min p.p. | 18 hours | Average |  | Costs standard method + Printing and postal costs extra letter: € 0.50 p.p. | € 1250 | Average |
| **4. Local media attention** | Standard time + Formatting article, addressing editors: 3 hours | Standard time | 13 hours | Average | Mostly free of charge | Costs standard method | € 938 | Average |
| **5. Reminder by SMS** | Standard time + Formatting and sending SMS: 3 hours | Standard time | 13 hours | Average |  | Costs standard method + Costs SMS for bundle: €0.20 p.p. | € 825 | Average |
| **6. Reminder by telephone** | Standard time | Standard time + Time for calling: 5 min p.p. | 41 hours | High |  | Costs standard method | € 750 | Average |
| **7. Pop-up reminders GP in computer system** | Standard time | Standard time + Entering pop-ups in system: 1 min p.p. + Addressing patients during consultation: 5 min p.p. | 16 hours + 31 hours for GP | High |  | Costs standard method | € 750 | Average |
| **8. Information gathering at the general practice** | Standard time + Organization for gathering: 8 hours | Standard time | 18 hours | Average |  | Costs standard method | € 750 | Average |
| **9. Self-management toolkits** | Standard time | Standard time + 10 min p.p. ordering a toolkit, time for mailing kits | 13 hours** | Average |  | Costs standard method + € 75 p.p. (costs kit + shipping costs) | € 2025 | High |
| **10. E-mail reminder increased risk patients** | Standard time + Formatting emails and mailing: 2 hours | Standard time | 12 hours | Average |  | Costs standard method | € 750 | Average |
